# Rural Suicide: Demographics, Causes, and Treatment Implications

**DOI:** 10.1007/s10597-024-01327-x

**Published:** 2024-08-05

**Authors:** Michael Prazak, Rachel Bacigalupi, Stephen C. Hamilton

**Affiliations:** Department of Counseling Psychology and Community Services, University of North Dakota, 231 Centennial Drive Stop 8255, Grand Forks, ND USA

**Keywords:** Rural, Suicide, Mental health

## Abstract

Suicide rates in rural areas are higher than urban areas and growing, with current treatment developments only exacerbating this discrepancy. Within individual factors, both age and gender relate to and intersect with personal values related to self-reliance and attitudes toward mental health difficulties and treatment to increase suicide risk. The lethality ubiquitous in rural environments and occupations is a leading factor in rural suicide rates, with other factors such as race alternately noted to be a key factor but with more mixed findings. The cultural values of rural communities as typically negative toward mental health disclosure and treatment contribute to the disengagement of rural communities from treatment that may otherwise prevent suicides, exacerbating the physical lack of treatment access many rural communities experience. Working within the primary care system alongside increased telehealth utilization are suggested to reduce rural suicide rates.

## Introduction

Though often marginalized and the subject of decreased clinical attention and services, the percentage of the American population considered rural is quite large. Estimates of the size of the rural population range from 46 million (Dobis et al., 2021) to approximately 60 million Americans and 97% of American land. Rural counties, with a typical population of 23,000, have significantly greater difficulty accessing healthcare, employment, and struggle with underdevelopment across indices. The implications of this are significant, with severe outcomes in mortality across the lifespan and healthcare system, including both physical (Moser et al., 2015) and mental health (Gamm et al., 2010).

Unemployment rates were typically very slightly higher than urban areas (4.4% vs. 4.1% in 2017), although urban areas benefit from faster employment growth than rural areas (Cromartie, 2018). That source, which represents a large study produced by the USDA, further reports issues of intersectionality in rural demographics, with 19% of the rural population 65 years old or older (compared to 15% urban), representing 85% of the national counties in America whose residents are 20% or greater 65 or older. This aging had further effects on employment rates, as labor force participation has declined, further exacerbating economic relocation of opportunities to urban areas. Rural areas are also somewhat less ethnically diverse – White residents are 80% of their population, vs. 58% of urban populations, with no ethnic minority group finding representational parity in comparison to urban areas. This is caveated by the finding that rural communities are slowly becoming more diverse (Rowlands & Love, 2021), although this has not yet received national recognition.

Consistent with relatively higher unemployment, rural poverty rate is significantly higher in rural (16.4%) than urban (12.9) areas, with widening disparity between the two and again similar points of overlap with other demographic factors. Specifically, African Americans showed the highest rural poverty rates, followed by Native Americans. Educational disparities also persist – rural adults are somewhat more than half as likely to have a bachelor’s degree as urban adults, with the gap widening markedly between 2000 and 2019 per the USDA (Marre, 2017), and much more likely to lack a high school diploma. This is thought to represent differences in income (25% less than urban households), physical distance to education institutions, and indicate much lower levels of academic achievement among African American and Hispanic rural individuals than rural White, with downstream effects in income and employment. The increasing age, decreasing employment, widening income gap within rural demographics and against urban areas, loss of youth and young adults, and increasing poverty create an increasingly disenfranchised, underserved, high-need, and marginalized subsection of American society.

One expression of the combination of demographic factors mutually contributing to a segment of society that is becoming increasingly disenfranchised, underserved, and struggling with precarious circumstances is that of high mental health needs, and low access to mental health treatment. Health disparities in rural vs. urban American populations have been studied across domains, but one in which resources and attention have been especially minimally allocated in response to persistent and pressing calls for services is that of mental health. Consistent with expectations, rurally-residing young adults were significantly less likely to be insured than urban communities (Chavez et al.^2018^), with nearly 20% of rural young adults with mental health concerns uninsured. The recognition of underserved mental health needs of rural Americans has been heard since at least the 1970s (Hollingsworth & Hendrix, 1977), with some initiatives in the 1980s to address this from the perspective of government-facilitated public health agencies and services (DeLeon et al., 1989).

Within the conversation on rural mental health needs, the area of suicide is one of particularly pressing urgency. Mental health disparities on the point of suicide, in fact, indicate only growing disparity favoring urban environments (Fontanella et al., 2015). Recent (2015) rates across rural American communities document suicide death rates at 19.93/100,000 for males from the age of 10–24 and 4.40 for females, approximately double the rates observed in urban communities. Young adults and adolescents in rural areas have demonstrated higher rates of death by firearm, suffocation and hanging than urban areas (Fontanella et al., 2015). In a British sample, similarly, suicide rates rose over the 10-year period studied most notably for women in rural areas (Middleton et al., 2003) with explanations given including reduced access to healthcare, barriers to help-seeking, economic difficulties, and mental health problems associated with rural locations.

## Literature Review

Despite the clear and pressing need, the disparity between rates of participation in the mental health system continue to grow (Patel et al., 2020), with rates of telehealth utilization in rural areas for bipolar disorder and schizophrenia increased over time, but not enough to compensate for decline in in-person mental health and less than the growth in urban areas. It has been observed that increased tele-mental health utilization during COVID increased, rather than decreased, care disparity for many rural areas (Summers-Gabr, 2020), exacerbating existing limitations to access for mental health care. In-person visits, predictably, are also indicative of limited access, with ambulatory mental health visits significantly less likely to be given when needed in rural areas, including those with ongoing psychiatric mental health treatment (Kirby et al., 2019).

### Causes

Exploring the specific factors causing increased rates of suicide in rural areas provides a path to determining treatment approaches, and ways of ameliorating risk factors while integrating care in ways most needed. After identifying, as have others, the rural-urban discrepancy in suicide rates, one Australian study (Inder et al., 2014) pointed to marital status, economic (employment, financial hardship), and psychological circumstances (distress, service use, service availability) as being suicide predictors, with demographics and age considered to be relevant to the findings. A qualitative study of rural Alaskan indigenous young adults (Skewes et al., 2022) identified mental health stigma, social changes, personal trauma, isolation, substance use and economic factors as relevant to suicide in their community. Economic/demographic, interpersonal (including broader social/community and relationship), psychological factors (mental health problems, including substance/alcohol, treatment stigma) and institutional barriers such as material treatment access barriers are each factors with unique relevance and features in rural communities that inform the discussion of suicide in rural areas.

Noting that rural Chinese communities demonstrate the same suicidal motiving factors as samples around the world, issues of physical and psychological health in combination with interpersonal concerns and life stressors each play a role in predicting suicidality (Zhang et al., 2004). Deficits in interpersonal, psychological and physical health have each been found as increasing rural suicide rates elsewhere (Manoranjitham et al., 2010). The combination of personal health, relationships, and environmental circumstances play a mutually exacerbating role in contributing to rural suicide.

### Personal

#### Age

Noting that older adults are the most likely to die by suicide across countries, one study of Canadian care home older adults (Neufeld et al., 2021) identified a mental health diagnosis, alcohol use/dependence, and loneliness as risk factors, as were rurality and age. The compounding effects of rural location on the difficulties of older adults including those related to increased substance and alcohol use, social isolation and mental illness are each important avenues of investigation. However, suicide in young adults in rural areas has also been studied extensively around the world, including in China where it leads death causes (Zhang et al., 2011a); Bangladesh (Begum et al., 2017); Australia, where rural suicide is disproportionately rising (Page et al., 2007); the US, where rural young adult suicides drastically outstripped urban and growing (Fontanella et al., 2015) and elsewhere (Mojs et al., 2012).

#### Gender

One study of suicide trends over 1970–1997 demonstrated that while the urban-rural differential rose over time in men, it somewhat diminished for women, indicating the possibility of a gender-rurality interaction in suicide method and factors (Singh & Siahpush, 2002). An Australian review (Alston, 2012) argued that for rural men, social factors, mental health stigma, method, and socioeconomic factors each interact with gender to contribute to uniquely high rates in rural males.

Consistent with studies from other areas, male gender is associated with higher lethality, with rural males but not females more likely to die by suicide than those in urban areas in one comprehensive review (Barry et al., 2020). The review did not study suicidality in terms of ideation or attempts. While male gender is consistently shown to relate to suicide death (Wexler et al., 2012) and hospitalization among rural populations across American communities, this tends to be a result of the more lethal means chosen by males (Kanchan et al., 2009) rather than rates of ideation, in which across ethnic groups females actually demonstrate higher rates of ideation and attempts (Langhinrichsen-Rohling et al., 2009). The interaction between male gender and firearm deaths in rural areas has been argued to account for much of rural-urban gender differences in suicide (Nestadt et al., 2017).

#### Race

Somewhat less-often identified as a significant contributor to rural suicide rates, race has also been argued to significantly account for much of the difference between rural-urban suicide rates (Peltzman et al., 2022), with rural Hispanic veterans demonstrating higher suicide rates than White or Black rural veterans. Various outcomes of the race-rurality interaction have been found, such as differences in suicide rates for specific ethnicities as a function of rurality, i.e. lower for rurally-residing black individuals compared to urban (Ivey-Stephenson et al., 2017). Although significant differences in suicidality according to race have not always been found (Nestadt et al., 2017), race is generally considered to be a significant factor in understanding rural suicide (Hirsch & Cukrowicz, 2014). Race has been argued to represent a “risk marker” rather than risk factor in previous literature (Brady et al., 2017), with white race in particular repeatedly identified as a unique risk marker of suicidality. Complicating the understanding of this factor are difficulties measuring race in relation to suicide in rural areas (McCarthy et al., 2012) and the community-specific, rather than global, relationship of race to suicide in a given area (Lopez-Castroman et al., 2015).

#### Method

Individuals in rural areas are significantly more likely to attempt suicide with firearms and hanging, and less likely to use poisoning (Barry et al., 2020). The observation that rural individuals are more likely to die by but somewhat less likely to attempt suicide is consistent with the conclusion that rural individuals use more deadly means for suicide attempts. The lethality of firearms as a major source of the higher rates of suicide in rural areas (Fontanella et al., 2015; McCarthy et al., 2012) was supported by follow-up examinations of lethality of suicide methods, particularly firearms, as a contributor to suicide rates (McKean et al., 2018). The greater availability of firearms in rural areas (Azrael et al., 2017) has been proposed as one explanation for the consistently higher firearm-based attempts, and thus total suicide death rate, so consistently found in rural areas (Conner et al., 2019). Although no identified literature has explored firearm storage in rural areas compared to urban, numerous articles have identified firearm storage as an important point of discussion (Betz et al., 2019; Johnson et al., 2004), and it is possible differences in this behavior do exist.

Potentially relevant to suicide lethality is the identified relationship between occupation, particularly agricultural (Arif et al., 2021; Kennedy et al., 2014) and economically precarious employment (Kalesan et al., 2020) to suicide rates. The lethality associated with traditionally-rural occupations may contribute to increased familiarity and comfort with more lethal means (Kohlbeck et al., 2022). This hypothesis has also been proposed and substantiated for other high-lethality occupations such as the medical field (Stark et al., 2006) Higher rates of gun ownership in rural areas (Kelley & Ellison, 2021), a necessity of rural lifestyles and many rural occupations (Stark, 2014), appears to both increase access to firearms (Skegg et al., 2010) and decrease barriers to use of firearms as a suicide method (Westefeld et al., 2016).

#### Financial

Financial difficulties experienced disproportionately in rural areas, as explored extensively above, may be one factor in the relatively higher rates of suicide in these areas. Within rural areas, socioeconomic variables such as marriage, income, and remoteness were associated with suicide rates (Berman, 2014). Employment status has been identified as particularly important in rural areas (Riva et al., 2011), a finding likely also the case in the context of mental health treatment resource disparities between rural-urban American communities (Brenes et al., 2015). Financial/circumstantial limitations in the sense of a disparity between expectations/potential and actuality have also been identified as suicide predictors in a rural Chinese sample of rural youth (Zhang et al., 2011a), consistent with the notion that an individual’s appraisal of circumstances may be as important as absolute circumstances, possibly outside the context of severe deprivation.

Conceptually related to financial circumstances in a broader way, temporary life circumstances, including transient but seemingly overwhelming stressors, may also lead to suicidality. This is the case for unemployment, related to suicidality in a rural sample (Taylor et al., 2004), along with education, an exacerbating factor in financial health. Adjustment disorders are related to rural suicide (Manoranjithamet al., 2010), an expected finding given the nature of this disorder as “distress that is out of proportion to the severity or intensity of the stressor (APA, 2022).”

#### Mental Health

Mental health disorders, including substance use specifically, are regularly identified as a core risk factor for rural suicide internationally (Pitman et al., 2012), as is psychological distress more broadly (Handley et al., 2012). While rural individuals struggling with suicide have been noted to have fewer mental health diagnoses and less treatment (Hirsch & Cukrowicz, 2014), this is likely a function of less access to mental health in this setting in general, rather than less psychological distress. The mental health disorders prevalent in rural areas, such as depression and anxiety (Gamm et al., 2010), do demonstrate some differences from urban areas, such as higher rates of these disorders and PTSD, but lower rates of bipolar disorder and schizophrenia (McCarthy et al., 2012). Rural individuals who died of suicide, consistent with other literature, were less likely to have a diagnosis, but with similar depression rates to urban individuals who had died of suicide (Searles et al., 2014). It has been noted that while depression relates to suicidality, the link between trauma and suicide risk is lower than that for urban individuals, partially attributed to possible underreporting.

### Interpersonal

From Durkheim’s foundational model of suicide as a function of social integration (Durkheim, 2005) through Freudian conceptualization of suicide as the end result of a lost object (Freud, 1917) to the leading contemporary model of suicide, the Interpersonal Theory of Suicide (IPTS) (Joiner, 2005), suicide has long been held to be largely a function of interpersonal health, both to other specific individuals and to society at large. For rural individuals, lower rates of social integration (Handley et al., 2012; Steelesmith et al., 2019) and social support (Handley et al., 2013) have been found to relate to higher rates of suicidal ideation.

Examining the social component of suicide in rural areas entails identifying those factors that, while not necessarily unique to rural areas, may be exacerbated by the characteristics of rural areas. Rural areas are described as having more traditional values (Waterson et al., 2016), with a focus on community (Howley & Howley, 2010) and self-reliance (Slama, 2004). While potentially at odds, the co-occurrence of self-reliance (as individuals, family structures, and specific communities) with community interdependence in rural communities has been documented extensively elsewhere (Binns & Nel, 2019). Similarly, they also demonstrate value differences toward mental health concerns and treatment in comparison to urban areas (Stewart et al., 2015). Rural suicide must be examined through the lens of sociocultural considerations (Carpenter-Song & Snell-Rood, 2017), without which intervention plans may be underutilized at best and harmful at worst. These considerations include financially precarious status with limited mobility opportunities, racial tensions and discrimination, and access inequalities, which may be partially mitigated by telehealth and government health and assistance programs.

A lack of perceived interpersonal support is consistently identified as a leading factor in suicidality (Runkle et al., 2022). Living alone and the loss of a stable romantic relationship in the last year have been identified as suicide risk factors in a rural Indian sample (Manoranjithamet al., 2010), consistent with American literature (Hirsch, 2006). Single marital status is also a risk factor for rural men in particular (Pitman et al., 2012). At one end of the spectrum of social support, peer victimization is a substantial suicide risk factor for rural youth (Bhatta et al., 2014) a finding which may contribute to understanding the suicide rates of rural LGBT individuals (Fisher et al., 2014; Poon & Saewyc, 2009), particularly in the case of social victimization due to sexual minority status (Harris, 2021). A social/community explanation is also given for elevated suicide rates among immigrant and racial minority communities (Pan & Carpiano, 2013), who face numerous other uniquely difficult circumstances in rural areas (Baffour, 2017).

### Barriers to Care

Identifying barriers to help seeking is a key imperative in reducing suicides in rural areas. Possible barriers include the material, such as lack of access to actual providers or health facilities; lack of health insurance or the means to pay for such services; and psychological barriers such as stigma and lack of information on how to receive mental health and the benefits of doing so.

#### Stigma

One qualitative study of rurally-residing veterans (Monteith et al., 2020) identified stigma about the discussion of suicide, corresponding infrequency of discussing such, and lack of knowledge/awareness as a result. The relationship between barriers is likely reciprocal: stigma leads to lack of knowledge, which inhibits healthcare utilization, which makes treatment less common, which prevents normalization, with each element exacerbating the others. One qualitative study of the identified barriers of rural veterans and mental health providers identified cultural attitudes of self-reliance and several other highly related cultural values as being primary barriers to help-seeking in rural communities (Fischer et al., 2016). Relatedly, a qualitative study of survivors following rural Canadian suicides (Creighton et al., 2017) identified barriers to communicating depressive symptoms in favor of self-medication in the context of perceived ideals of masculinity and the experienced inability to seek psychological help as a rural man, an attitude which is a key contributor of rural mental health stigma. Similarly, while demonstrating statistically equivalent rates of mental illness, rural men sought mental healthcare at lower rates than urban counterparts (Caldwell et al., 2004). Although literature contrasting attitudes toward masculinity in rural vs. urban populations is scarce (Silva, 2022), significant differences have been identified. These include increased self-reliance of rural men in types of labor (Moisio et al., 2013), increased legitimization of violence (Carrington & Scott, 2008), and self-stigma (related to help-seeking) double the size among rural vs. non-rural males (Hammer et al., 2013).

#### Lack of Access

The rural south has long been known as a region with drastic scarcity of mental health services, with general medical facilities rather than specialty mental healthcare often serving as the only source of psychological treatment (Fox et al., 1995). Overreliance on medical professionals who are not trained in specialty mental health in the context of high demand and low supply for mental health services is one consistently found domestically and internationally (Beks et al., 2018). Although the lack of any psychiatric care in many rural areas is one common example of lack of providers as a barrier to healthcare (Fortney et al., 2015), the explanation that rural suicide rates are reflective of lack of provider availability has been challenged (Fiske et al., 2005). Despite this, the association of fewer mental health providers in a rural area with higher suicide rates has generally been found (Graves et al., 2020). The provision of mental health through non-specialty providers may exist, but improvements in knowledge and familiarity for this cross-training is also routinely suggested (Smalley et al., 2010).

One article exploring barriers to family mental health treatment identified access limitations and broader availability concerns alongside social stigma as issues to navigate (Jensen & Mendenhall, 2018). This is similar to other findings, which have found various dimensions of access limitations to mental health treatment in rural areas (Murray-Swank et al., 2018). The combination of higher mental illness rates in rural areas that also have lower treatment access is a problem around the world (Jagodic et al., 2012).

#### Insurance

Clear differences in rural-urban rates of health insurance persist (Day, 2021), with critical implications for treatment participation. Rural areas demonstrate higher prescription but lower therapy engagement (Ziller et al., 2010), with this finding framed in the context of affordability and insurance disparities. Rural residents are significantly more likely to treat depression pharmacologically rather than with psychotherapy (Fortney et al., 2010), with this finding mediated by psychiatrist availability. Further, it was noted that rural psychotherapy demonstrated decreased adequacy in comparison to urban areas. Rural residents pay higher out-of-pocket costs for mental health and demonstrate corresponding lower number of visits received vs. primary care (Chen et al., 2022). Uninsured individuals in rural areas have unmet mental health needs five times higher than the insured (Roll et al., 2013). Rural areas are also more likely to lack Medicaid-accepting mental health agencies (Cummings et al., 2013).

### Treatment

Despite decades of federal attention to the need to provide mental health treatment in rural areas (Human & Wasem, 1991), the need persists. Presently, diversifying approaches to mental health treatment, away from traditional specialty providers and toward increased points of access and appropriate training appears useful. As one example, increased access to independently-practicing PMHNPs in combination with telepsychiatry has been shown to effectively bring increased services to rural areas (Finley, Myers, 2020). The identification of telehealth as a major pillar of rural mental health is a commonly identified one (Myers, 2019), with COVID likely only catalyzing this. The utility of telehealth for rural individuals has clear benefits, with corresponding attention to this treatment modality as a result (Lu et al., 2014).

Noting that few treatment resources are specifically targeted toward either veterans or rural individuals, with none for the unique subset of both, one scoping review of treatment options (Mohatt et al., 2018) identified findings related to improved access, primary care, community training and awareness as each contributing to treatment in underserved areas. Programs that do exist to address and introduce rural mental health treatment seem focused largely in the primary care setting (McFaul et al., 2014). For minors, integrating suicide treatment into the existing school mental health system seems to be one feasible way to expand toward suicide prevention (Schmidt et al., 2015).

Consistent with the notion that suicide has both individual and societal/cultural factors, treatment recommendations have also been addressed dually to each, encouraging an integration of cultural factors that may otherwise represent suicide risk factors (Taylor et al. 2014). Similarly, community-based approaches integrating a variety of methods to reduce suicide from a social orientation have preliminary success (Walker et al., 2019), consistent with the established social basis of much suicidal behavior (Levi-Belz, 2019). Combining technological approaches to overcome the numerous institutional access barriers of rural areas to address mental health needs from a peer-based social perspective, pilot studies have also begun to develop programs that would decentralize peer support to increase community mental health support (Pisani et al., 2018).

### Implications

The problem of suicide in rural mental health areas is a serious concern with significant and growing discrepancies between rural and urban suicide mental healthcare. Relevant individual factors, such as age, gender, race, access to lethal means, and financial difficulties were each identified. Social factors such as lack of interpersonal support, social victimization, and socially influenced underreporting of mental health disorders each lead to high rates of rural suicide. Numerous possible and proposed barriers to rural healthcare engagement exist, including stigma (Wrigley et al., 2005), physical access barriers such as proximity/distance (Parr et al., 2004), and financial resource lack/economic underdevelopment (Larrison, 2011).

Recognizing the numerous barriers to mental health treatment, primarily including attitudes against mental health treatment, and decreased access (both in terms of availability and financial means), structuring assessment and intervention to destigmatize mental health difficulties, increase willingness to engage in self-disclosure, and work within the confines of limited mental health treatment availability are imperative. Despite telehealth repeatedly identified as a leading option to increase availability, the lagging rates of telehealth participation relative to urban areas indicate this is not a complete solution, and strategies to improve telehealth access as well as normalize this and broader treatment within rural communities would be helpful.

Assisted Outpatient Treatment (AOT), a program of court-ordered outpatient treatment, has proven promising but with some challenges and corresponding calls for support (Cripps & Swartz, 2018). AOT has been applied in both urban and rural populations (Torrey & Zdanowicz, 2001) with positive outcomes in reducing violence. AOT demonstrates differences in utilization across states (Medrum et al., 2016), but may prove promising in overcoming some cultural barriers to mental health treatment in a population that poses unique risks related to suicidality. Similarly, Assertive Community Treatment (ACT) has been explored in rural communities via telehealth (Swanson & Trestman, 2018), with accommodations for the challenges of ACT in rural areas noted (Trane et al., 2022). ACT represents a multidisciplinary model to direct patient care and has shown success in rural populations specifically (Meyer & Morrissey, 2007).

Given the relatively higher rates of reliance on primary care as a sole mental health treatment approach, one primary implication is the importance of improved screening and awareness of mental health, and suicidality in particular, in this setting. In the context of stigma in rural communities against mental health disclosure and treatment, structuring assessment to fully capture life stressors, particularly financial, as well as interpersonal difficulties such as peer or social victimization may also provide warning signs of increased risk. With the relative lack of stigma of medical utilization as opposed to specialty mental health, utilizing primary care as a way of addressing mental health treatment and working within the attitudes of rural communities is consistent with some recommendations from within this field (Shim & Rust, 2013). Changing rural attitudes toward mental health, while valuable, is likely less immediately feasible than increasing the availability and quality of care within the primary setting to address existing mental health needs. However, coupling this with increased availability and normalization of telemental health treatment, increasing awareness of suicide risk factors, and more comprehensive treatment across modalities are each suggested from the literature.
